# In vitro maturation (IVM) of human immature oocytes: is it still relevant?

**DOI:** 10.1186/s12958-023-01162-x

**Published:** 2023-11-22

**Authors:** Mausumi Das, Weon-Young Son

**Affiliations:** 1 Department of Reproductive Medicine, Queen Charlotte and Hammersmith Hospitals, Imperial College Healthcare NHS Trust, London, UK; 2 Chelsea and Westminster Hospital NHS Foundation Trust, London, UK; 3 Alreem Medical Center, Muscat, Oman

**Keywords:** Fertility preservation, Immature oocytes, In vitro maturation (IVM), Maturation mechanism, Ovarian Hyperstimulation syndrome (OHSS)

## Abstract

In vitro maturation (IVM) of human immature oocytes has been shown to be a viable option for patients at risk of ovarian hyperstimulation syndrome (OHSS), those seeking urgent fertility preservation and in circumstances where controlled ovarian stimulation is not feasible. Moreover, IVM techniques can be combined with ovarian tissue cryobanking to increase the chances of conception in cancer survivors. The clinical applications of IVM in the field of reproductive medicine are rapidly expanding and the technique is now classified as non-experimental. In contrast to conventional IVF (in vitro fertilization), IVM offers several advantages, such as reduced gonadotropin stimulation, minimal risk of ovarian hyperstimulation syndrome (OHSS), reduced treatment times and lower costs. However, the technical expertise involved in performing IVM and its lower success rates compared to traditional IVF cycles, still pose significant challenges. Despite recent advances, such as innovative biphasic IVM systems, IVM is still an evolving technique and research is ongoing to refine protocols and identify techniques to improve its efficiency and effectiveness. A comprehensive understanding of the distinct mechanisms of oocyte maturation is crucial for obtaining more viable oocytes through in vitro methods, which will in turn lead to significantly improved success rates. In this review, the present state of human IVM programs and future research directions will be discussed, aiming to promote a better understanding of IVM and identify potential strategies to improve the overall efficiency and success rates of IVM programs, which will in turn lead to better clinical outcomes.

## Background

Oocyte In vitro maturation (IVM) is a technique in assisted reproductive technology (ART) that involves the maturation of immature oocytes obtained from small antral follicles. Since the first IVM baby was born in 1991 [1], the use of IVM has been credited with approximately 5000–6000 live births [2]. In recent years, IVM has gained popularity in the field of reproductive medicine, and it is being used for carefully selected patients at risk of ovarian hyperstimulation syndrome (OHSS) or seeking fertility preservation. The clinical applications of IVM have expanded to include patients experiencing repeated failures in assisted reproduction due to resistant ovary syndrome and poor responders [3–5]. Recently, the American Society of Reproductive Medicine (ASRM) has acknowledged the usefulness of IVM in certain clinical applicationsand has declared it to be nonexperimental [6]. Overall, IVM of human immature oocytes remains a relevant and valuable technique in the field of assisted reproduction and fertility preservation.

The complexity involved in performing IVM and its generally lower success rates compared to conventional IVF remain significant challenges [7–9]. Additionally, advancements in antagonist cycles, GnRH agonist triggering, and elective cryopreservation strategies have led many IVF centers to prefer easier treatment methods for infertility patients, resulting in a limited number of centers currently performing the IVM procedure.

The lower efficiency of human IVM programs can be attributed to the quantity and quality of oocytes obtained during IVM cycles. Firstly, the rate of oocyte retrieval and IVM after in vitro culture is lower compared to conventional controlled ovarian hyperstimulation (COH) cycles. Secondly, the overall quality of oocytes derived from current IVM culture systems tends to be inferior to naturally matured oocytes in vivo [10]. Therefore, ongoing research and improvements of the IVM program are necessary to offer valuable solutions and additional options to individuals facing fertility challenges.

In this review, the present state of human IVM programs and future research directions will be discussed, aiming to promote a better understanding of IVM and identify potential strategies to improve its efficiency and success rates.

## Methods

This review is grounded in content obtained through electronic search of PUBMED. It encompasses articles related to IVM published in the English language.

### Oocyte maturation

A Comprehensive understanding of the distinct mechanisms of oocyte maturation both in vivo and in vitro environments is crucial for obtaining more viable oocytes through in vitro methods.

The maturation of oocytes can be divided into two essential aspects: nuclear maturation and cytoplasmic maturation [11]. Nuclear maturation primarily involves the process of meiosis, which is responsible for the reduction of the chromosomal number in the oocyte. It starts from the Prophase-I stage (germinal vesicle (GV-) stage) where the oocyte is arrested. Meiotic resumption is characterised by GV breaks down (GVBD), chromatin condensation and the formation of the meiotic spindle. The oocyte then undergoes the first meiotic division, leading to the extrusion of the first polar body and the formation of a mature metaphase II (MII) oocyte, which has a haploid chromosomal complement (Table 1). At this stage, the oocyte is arrested again, and it awaits fertilization [11].

**Table 1:** Overview of the alterations of nuclear and cytoplasm during oocyte maturation

Nuclear maturation Stage	**GV (Germinal Vesicle)**	**GVBD (Germinal Vesicle Breakdown)**	**Metaphase-I**	**Metaphase-II**
Cytoplasmic Organelles	Prophase-I	Nucleus of the oocyte breaks down and the nuclear envelope disintegrates	The chromatin within the nucleus condenses (Chromosome condensation)	The first polar body extrusion (chromosome segregation) and the chromosomes align at the metaphase plate
Mitochondria	Aggregated around the GV Predominantly spherical to oval with dense matrices and few arch-like or transverse cristae presenting an inert appearance Usually absent from the cortical part of the cytoplasm	Move away from the perinuclear region Upon GVBD, most mitochondrial clusters are larger. Later, a large proportion form smaller clusters throughout the cytoplasm	More numerous and dispersed in the ooplasm	Occupy most of the egg volume Voluminous aggregates with SER tubules and vesicles (human)
Golgi apparatus	Dispersed throughout the ooplasm in the form of a continuous membranous system, but it is slightly more concentrated in the interior than at the cortex	Fragmented and aggregated in the central part of the oocyte	Further fragmented and dispersed throughout the oocyte	The distribution is maintained
Endoplasmic reticulum (ER)	Uniformly distributed throughout the cytoplasm	Localizes in cortical regions	Concentrated in the vegetal half of the mature egg	ER clusters are present throughout the entire oocyte
Cortical granules	Uniformly distributed throughout the cytoplasm	Uniformly distributed throughout the cytoplasm	Migrate towards the cortical cytoplasm and arrest in the cortex	Arrest in the cortex Present over the spindle region (human)
Cytoskeleton:				
- Intermediate Filament	Confined to large cortical aggregates	Confined to large cortical aggregates	Disperse into multiple small spots	Evenly distributed throughout the cytoplasm
- Microtubules	Uniformly distributed throughout the cytoplasm	Condensed around the chromosomes	Observed as fully organized meiotic spindles	Observed as fully organized meiotic spindles
- Microfilament	Uniformly distributed throughout the cytoplasm	Densely accumulated in the subcortical region of the oocytes	Densely accumulated in the subcortical region of the oocytes	Mainly accumulate in the cortical cytoplasmic region

*Abbreviations: SER* Smooth endoplasmic reticulum

Edited from L Mao et al. [12]

Cytoplasmic maturation is a vital process occurring concurrently with nuclear maturation in the oocyte. It involves metabolic and structural changes, including the accumulation of factors and rearrangement of organelles like mitochondria and the endoplasmic reticulum [11, 12] (Table 1). These changes ensure proper support for fertilization and embryo development. Synchronization of both nuclear and cytoplasmic maturation is crucial for successful fertilization and the healthy development of the embryo [11].

### In vivo oocyte maturation

In vivo, oocyte maturation is regulated by hormonal signals, interactions with somatic cells, and transcription factors controlling gene expression [13]. In a regular menstrual cycle, one dominant follicle develops into a preovulatory follicle, where the oocyte remains arrested at Prophase-I stage until the surge of ovulatory luteinizing hormone (LH) [11]. This meiotic arrest is maintained by high levels of intracellular cyclic adenosine 3', 5'-monophosphate (cAMP) within the oocyte, keeping it at the GV-stage [14–20]. The communication between the oocyte and cumulus cells (CCs) through gap junctions is crucial for regulating oocyte maturation [21]. Throughout follicle growth, this communication is essential for providing nutrients, energy substrates, and factors that support oocyte maturation and developmental competence [21].

Animal research has identified three mechanisms that help maintain high cAMP levels within the oocyte, regulating oocyte maturation and meiotic progression: (1) The oocyte itself produces cAMP [15, 16], (2) cAMP produced by CCs enters the oocyte through gap junctions [21], and (3) cGMP produced by granulosa cells passes through gap junctions into the oocyte [17–20] and inhibits the hydrolysis of cAMP by oocyte-specific phosphodiesterase (PDE) [22, 23]. Guanylate cyclase receptor natriuretic peptide receptor 2 (NPR2) mediates cGMP production in granulosa cells through the action of their ligand C-type natriuretic peptide (CNP) [24]. The elevated intra-oocyte cAMP concentration leads to high protein kinase A (PKA) activity, which phosphorylates cell cycle components, including the meiosis-promoting factor (MPF), ultimately blocking meiotic progression.

Following the LH surge, oocyte maturation in vivo is initiated through cascade signalling pathways and physiological changes within the preovulatory follicles. LH activation of mural granulosa cells leads to the expression of epidermal growth factor (EGF)-like growth factors such as betacellulin, amphiregulin (AREG), and epiregulin [25–27]. These EGF-like growth factors bind to their receptors in CCs and activate the mitogen-activated protein kinase (MAPK) pathway. The activation of MAPK induces the synthesis of meiosis resumption-inducing factors and impedes the functioning of gap junctions. Simultaneously, the LH surge deactivates NPR2 and activates cGMP PDE, causing a rapid decline in cGMP levels within the follicle [28]. This decrease in cGMP supply to the oocyte leads to a subsequent drop in cAMP levels, inactivation of PKA, dephosphorylation of key components, and the initiation of meiosis.

Physiologically, the LH surge triggers the enlargement of the Graafian follicle. The surrounding CCs undergo expansion, crucial for oocyte maturation. Hyaluronan (HA) synthesized by Hyaluronan synthase 2 (HAS2) in CCs plays a significant role in this process [29, 30]. Soluble factors like differentiation factor-9 (GDF-9), morphogenetic protein 15 (BMP-15), and BMP-6, produced by the oocyte, actively participate in HA synthesis and CCs expansion [31, 32]. These factors stimulate HAS2 gene expression, promoting CCs expansion in the presence of FSH [31, 32]. The expanded CCs disrupt gap junctions in the COCs, stopping cAMP and cGMP transport, leading to oocyte meiotic resumption via MPF activation [33]. These coordinated molecular signals ensure successful oocyte maturation within the follicle.

### Laboratory and clinical aspects of IVM

#### In vitro oocyte maturation

Compared to the natural oocyte maturation process that occurs in vivo, IVM lacks the signalling mechanisms responsible for maintaining oocyte arrest in Prophase-I stage. This is due to the extraction of oocytes from the follicular environment, leading to the loss of influences including CNP from somatic cells and follicular fluid. Consequently, immature oocytes retrieved from antral follicles in vitro undergo spontaneous meiotic maturation independent of hormonal regulation. This spontaneous maturation leads to the premature breakdown of gap junctions between the oocyte and CCs. As a result, valuable CCs metabolites such as nucleotides, nutrients, and mRNA, which play a role in oocyte cytoplasm maturation, are lost. This factor represents a significant barrier to the generation of high-quality embryos from IVM oocytes.

The conventional approach to IVM involves culturing immature COCs from the Prophase-I to reach the metaphase II (MII) stage without the administration of any gonadotropins [34]. However, in clinical human IVM programs, it is common to use in vivo stimulation with gonadotropins to improve the quality and quantity of oocytes. This stimulation can include a few days of gonadotropin (FSH) treatment, a single ovulatory dose of human chorionic gonadotropin (hCG), or a combination of FSH and hCG [34].

### FSH priming

Ovarian stimulation with a few days of FSH priming is often used in clinical IVM programs. Animal studies have suggested that in vivo FSH priming enhances follicular development and the meiotic and developmental competence of immature oocytes and decreases the time required to reach the MII stage [35, 36]. Likewise, in human IVM programs, FSH priming has been found to improve oocyte yield and maturation rates, resulting in more mature oocytes. The rationale for pretreating a patient with FSH is that human follicles with a diameter of 2–6 mm have a high expression of FSH receptors, and FSH augments follicular growth and estradiol production.

**Fig. 1:**
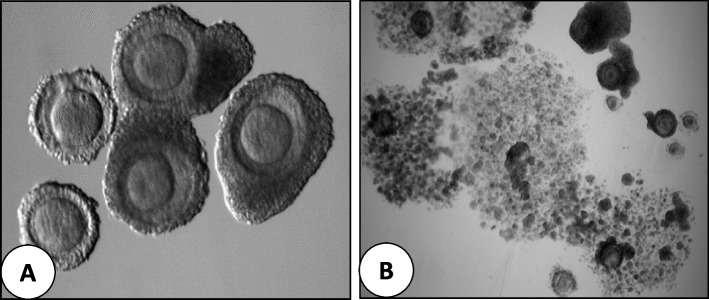
Cumulus-oocyte complex (COC) retrieved from FSH- or hCG-primed IVM cycles. **A** Oocytes just after oocyte retrieval in FSH-primed IVM cycles (Original magnification X100). **B** Oocytes just after oocyte retrieval in hCG-primed IVM cycles. (Original magnification X20)

As FSH priming does not induce oocyte meiotic resumption in vivo, immature compact COCs are obtained after oocyte retrieval [37] (Fig. 1A). There is no consensus, however, on the dose and duration of FSH priming in IVM cycles. Wynn et al. [38] suggested that a short course of FSH treatment (600 IU for 5 days, from day 2 of the menstrual cycle), improved the oocyte maturation rate in vitro. In a small, randomized study in 28 women with PCOS, the percentage of oocytes reaching the MII stage was significantly higher in women who had undergone FSH priming (150 IU recombinant FSH for 3 days, starting on day 3 of the cycle), compared with the non-primed group [39]. Other authors have also reported using 150 IU FSH daily for 2–3 days, starting from day 2 or 3 of the cycles or after a progestin withdrawal bleed [40].

Despite the rationale of FSH priming in IVM, FSH priming alone without hCG did not result in improved clinical outcomes in women without PCOS [41]. Likewise, Mikkelsen et al. [42] found that FSH priming did not improve the maturation potential of in vitro matured human oocytes. In PCOS patients, Mikkelsen and Lindenberg [43] suggested that FSH priming may improve the oocyte developmental competence whereas other authors did not find any significant differences [44, 45].

### hCGpriming before oocyte retrieval

Researchers have suggested that hCG may promote the initiation of oocyte maturation in vivo and improve the maturation rate of IVM oocytes, thereby improving pregnancy rates. In conventional IVF cycles, the maturation of immature prophase-I stage oocytes, especially those with expanded CCs, tends to be more successful. The integration of hCG priming in IVM cycles aims to mimic the conditions seen in conventional IVF cycles, where expanded CCs correlate with better maturation rates.

In a prospective randomized study in patients with PCOS, the percentage of oocytes achieving maturation at 48 h was significantly higher in the hCG primed group than in the non-hCG primed group [46]. Conversely, other studies did not find any significant differences in developmental competence of the oocytes or in clinical outcomes [47]. Additionally, Fadini et al. [41] reported that hCG priming alone has no beneficial effect on the clinical outcome in patients without PCOS.

**Fig. 2:**
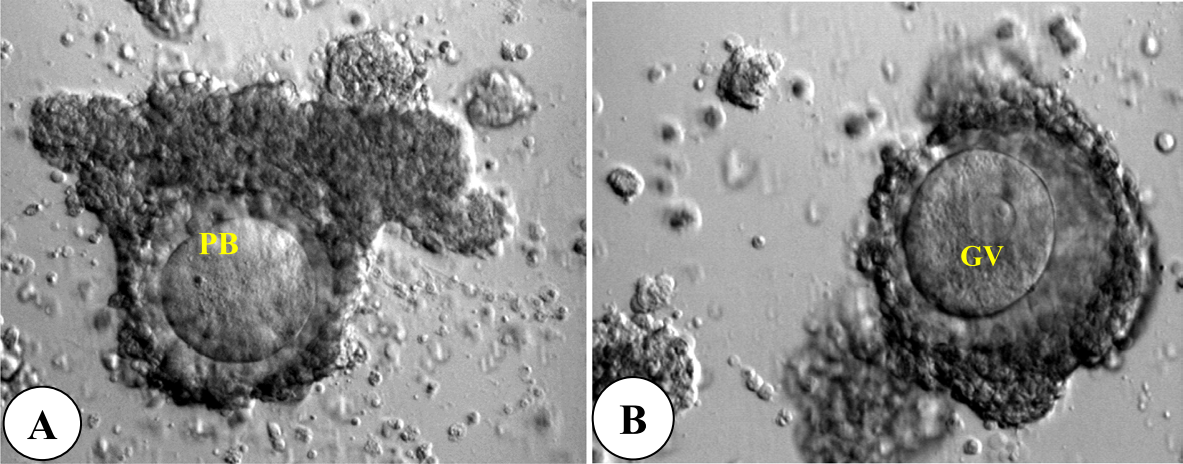
Cumulus − oocyte complex morphology of oocytes with expanded cumulus cells (CC) collected from < 10mm size of follicles in hCG-primed IVM cycles. **A** Metaphase II (MII)-stage oocyte with little expanded corona radiata. **B** Germinal vesicle (GV)-stage oocyte with little expanded corona radiata. Original magnification (× 200). PB = 1st polar body

Several studies have described hCG triggering combined with FSH priming in IVM cycles with variable outcomes. Lin et al. [48] did not observe any additional benefit from FSH priming in hCG-primed IVM cycles in PCOS women. However, Fadini*et al*. [41] in a prospective randomized study, reported a significantly higher clinical pregnancy rate in FSH plus hCG primed cycles, although FSH priming and hCG priming alone showed no significant differences in clinical outcomes. In a large retrospective cohort study of 921 women with PCOS who underwent IVM cycles with FSH priming with hCG triggering, the authors reported a cumulative live birth rate of 33.7% after one IVM cycle [49]. Son et al. [45] observed that in vivo matured oocytes can be collected from small follicles measuring < 10 mm at the time of oocyte retrieval (Fig. 2) and sometimes more than one in vivo matured oocyte can be retrieved in hCG-primed IVM cycles of PCO patients [50]. Furthermore, the authors reported that the matured oocytes retrieved from small follicles (< 10 mm) generated embryos of similar developmental potential to oocytes derived from larger follicles (≥ 10 mm), resulting in better pregnancy rates. However, a recent Cochrane review found no conclusive evidence that hCG triggering before oocyte retrieval in IVM cycles influenced live birth, pregnancy or miscarriage rates, although the quality of evidence was low [51].

Although the use of a GnRH agonist trigger before oocyte retrieval has been described in a case report [52], more evidence is needed before it can be routinely used in clinical practice.

Initially, since antral follicles are mostly under 12 mm, assessing in vivo matured oocytes on collection day was omitted [46]. Granulosa cells lacked LH receptors, signalling incomplete in vivo maturation. Yet, Son et al. [45, 53] identified in vivo mature oocytes via hCG priming, highlighting their high reproductive capacity [34, 45, 53]. This finding has led to debate among scientists about the role of hCG in IVM programs [54, 55].

### Timing of oocyte retrieval

Selection of the optimal day for oocyte retrieval in IVM cycles differs widely between groups. While some investigators have proposed waiting for the leading follicle to reach 10 mm [42, 56], others believed that this would be detrimental and suggested cancelling the cycle [57, 58]. In a study involving 160 women with polycystic ovaries, Son et al. [50]concluded that oocyte collection in IVM cycles should be performed when the dominant follicle is 14 mm in a diameter or less as sibling immature oocytes may be affected adversely if a dominant follicle > 14 mm is present at the time of oocyte retrieval.

Researchers have also suggested that extending the period of hCG priming time from 35 to 38 h for immature oocyte retrieval promotes oocyte maturation in vivo and increases the IVM rate of immature oocytes [59].

Clearly, further evidence from well-designed RCTs is needed to confirm the benefits of priming with gonadotropins on small follicles in IVM cycles, and the optimal dose and timing. As IVM programs are limited to a few specialized centers around the world, an international registry would facilitate data sharing and would encourage best practice.

### Oocyte retrieval

Unlike in standard IVF collections, oocyte retrieval in IVM cycles can be more challenging, due to smaller follicle sizes and stronger attachment of immature COCs to the follicle wall, especially without an ovulatory dose of hCG or GnRH agonist. Emphasizing the importance of correctly timed ovulating hCG injections in IVF cycles is vital, as retrieval may fail even from larger follicles without them. Several published articles have demonstrated that cycles with a higher number of immature oocytes retrieved tend to yield better clinical outcomes [60]. Hence, achieving a satisfactory oocyte recovery rate is crucial for the success ofIVM programs.

In the majority of IVF centers, the standard needle diameter for puncturing small follicles, which can range from 2 to 12 mm in size, is typically between 16 and 21 gauge. However, some clinics employ a two-needle system for follicle aspiration in IVM cycles. The reported aspiration pressure varies from 80 to 120 mmHg [2].Using a thin needle for multiple punctures on small follicles, along with lower aspiration pressure, may occasionally result in needle blockage during the procedure [34]. To prevent the formation of blood clots inside the aspiration needle, regular flushing with flushing media or adding 2 U/mL of heparin is necessary. This precaution helps maintain the patency of the needle and ensures smooth aspiration without interruptions caused by clot formation.

A retrospective cohort study comparing complication rates and pain scores after oocyte retrieval in IVM and IVF cycles concluded that although IVM oocyte retrievals require more punctures per ovary and took significantly more time than IVF oocyte collections, they were not associated with a higher complication rate than IVF oocyte retrieval procedures [61].

When directly aspirating immature oocytes from ex vivo ovarian tissues or ovaries during a caesarean section (CS), a simple technique is commonly used. The tissues or ovaries are held with one hand, while a 5 to 20 mL syringe filled with buffered IVF media containing proteins is used with a 21- or 22-gauge needle for the aspiration process [62, 63]. This technique allows for efficient retrieval of small follicles containing immature oocytes.

The identification of oocytes from follicular aspirates in IVM cycles can be approached using two methods: the direct method and the filter method [34]. In the direct identification method, the follicular aspirate is transferred to a Petri dish and examined under a stereomicroscope to detect COCs, similar to the process in conventional IVF cycles. This approach is employed in both hCG-primed cycles, with or without priming with FSH, as many of the retrieved oocytes have expanded CCs [34](Fig. 1B).

However, since identifying immature oocytes lacking an expanded cumulus mass in non-hCG primed IVM cycles can be more challenging than in IVF cycles, the filter method is commonly used in many IVF laboratories. The filter method involves using a cell strainer device with 70-μm pores, usually composed of a nylon mesh (such as Falcon® 70 μm mesh size from BD Biosciences) [34]. In this method, the follicular aspirates collected in tubes are passed through the cell strainer, which helps separate the oocytes from other components. Once the aspiration is complete, the collected aspirates on the device are rinsed with fresh buffered medium to eliminate red blood cells and small cells. The oocytes, along with other components like granulosa cells, are then transferred to a new Petri dish for examination under a stereomicroscope to identify and isolate the COCs. While the filter method is efficient and straightforward, it may lead to a delay in communication between the clinician and embryologist regarding the quantity and morphology of the retrieved oocytes during the process. Clinicians often want to know the number of oocytes obtained during the egg retrieval procedure to assess the progress of the aspiration process.

### IVM of immature oocytes, culture medium for IVM and supplements

As mentioned earlier, the spontaneous maturation of immature oocytes in vitro can lead to incomplete cytoplasm maturation, potentially impacting the developmental competence of the oocytes and resulting embryos. To address this issue and improve the overall success of IVM programs, some studies have explored a biphasic IVM approach, which involves two phases: pre-IVM and IVM. In the pre-IVM phase, chemicals such as cAMP analogues, kinase inhibitors, or PDE inhibitors are used to delay or temporarily prevent spontaneous oocyte maturation for a short period (usually around 2 h) [64]. However, the effect of using these chemicals in humans has not yielded significant improvements in outcomes.

Recently, a novel biphasic IVM method has been reported, which aims to mimic the in vivo system. In this method, CNP is utilized to maintain GV arrest for 24 h in the pre-IVM phase. As mentioned earlier, CNP regulates cGMP levels in granulosa cells inside the follicle. COCs treated with CNP sustain gap-junctional communication between the oocyte and CCs for a longer duration, supplying the cGMP produced to the oocytes, even in in vitro conditions [65]. This innovative biphasic IVM system has been termed 'CAPA-IVM' [66], and studies have reported highly promising outcomes [67]. It is worth noting that this culture system requires an extra day of culture and has only recently been introduced into clinical practice in an IVM laboratory. Consequently, its efficiency still needs to be confirmed by other IVF centers to validate its potential benefits and success rates.

Indeed, the majority of clinics perform IVM without utilizing the pre-IVM phase, and there is no consensus on the most suitable medium formulation for IVM [66, 67]. Although basic human IVM culture media are commercially available, the developmental competency of immature oocytes was not significantly different when using complex culture medium or regular IVF media such as blastocyst media which has a high concentration of glucose [68, 69].

Currently, many IVM protocols involve adding serum, FSH, LH/hCG, or EGF-like growth factors to the culture medium based on their role in oocyte maturation in vivo. To avoid potential risk ofinfection from serum sources, alternatives like the patient's own serum, human serum albumin, or synthetic substitutes have been used for protein supplementation in IVM [70].

FSH is commonly included in the culture media during IVM to support oocyte maturation. In vivo, FSH is crucial for the development of preovulatory follicles, the induction of LH receptors, and the stimulation of EGF-like growth factors [71, 72]. Similarly, LH and hCG are essential in IVM media to mimic the natural process of meiotic resumption and final maturation after LH surge and ovulation in vivo [73–75]. However, it is essential to note that the role of gonadotropins in vitro may differ from in vivo due to the absence of mural cells, which mediate LH/hCG signals and EGF-like growth factors, in immature COCs collected in vitro. Therefore, one study suggested using high FSH levels (70 IU/L) in IVM to stimulate the somatic cells of COCs, which have relatively low levels of FSHR expression, to sufficiently secure LHR expression, thereby enabling oocytes to resume meiosis [76].

Recent IVF research has explored the addition of EGF-like growth factors (AREG or/and epiregulin) [77], midkine [78], oocyte-secreted factors (GDF-9, pro-GDF9, BMP-15, or pro-BMP15) [79, 80], cumulin [81], or antioxidants (melatonin or coenzyme Q10) [82–84] to IVM media, resulting in improved maturation and embryo development. Cadenas et al. [76] confirmed significant up-regulation of substances such as amphiregulin, inhibin-A, inhibin-B, and midkine in human follicular fluid (FF) and granulosa cells (GCs) during the final maturation of follicles in vivo.

However, further extensive research is necessary to determine the optimal combination and concentrations of these supplements in the culture medium to achieve enhanced IVM outcomes.

### IVM culture time and Insemination

A critical aspect of the hCG-primed IVM cycles is the identification of in vivo mature oocytes on the day of collection, attributed to their significant reproductive potential (Fig. 2). Consequently, in hCG-primed cycles, it is crucial to assess oocyte maturity multiple times, encompassing both the collection day and the subsequent day. This is in contrast to other non-hCG primed IVM programs, where in vivo matured oocytes are not present on the collection day.

In the early IVM studies, oocyte maturity was typically assessed after 48 or 56 h of culture [1, 85, 86]. However, recent researchers have shown that a significant number of MII stage oocytes (approximately 40–60%) can be obtained after just one day of culture (approximately 24–30 h) from GV stage oocytes collected in IVM cycles [34].

Historically, intracytoplasmic sperm injection (ICSI) has been the preferred method of insemination in IVM studies [60, 87]. While IVF fertilization methods can be used in IVM [88, 89], ICSI is commonly used to increase fertilization rates, regardless of the presence of a male factor, and to mitigate the risk of unexpected fertilization failure.

### Culture of IVM embryos, Embryo Transfer (ET) and cryopreservation

After zygotes (fertilized oocytes) are generated through IVF fertilization or ICSI within an IVM cycle, the subsequent embryological tasks and procedures for ET and cryopreservation closely resemble those employed in traditional IVF cycles [34].

Although the cleavage rate of IVM embryos parallels that of IVF embryos, the blastocyst rate of IVM embryos is generally somewhat lower than that observed in IVF embryos [90, 91]. Consequently, numerous IVF centers initially leaned toward cleavage stage ET in IVM cycles to avert potential risks linked to difficulties in blastocyst development. Nevertheless, certain centers have endeavoured to enhance clinical outcomes by cultivating and transferring IVM embryos at the blastocyst stage, resulting in improved success rates [7, 91].

In terms of cryopreservation, investigations have revealed favourable survival rates and reasonable clinical outcomes when employing vitrification to freeze both cleavage-stage and blastocyst-stage embryos derived from clinical IVM programs [92–94].

### Endometrial preparation and luteal support

In IVM cycles, adequate endogenous estrogen from the dominant follicle to prepare the endometrial lining is deficient and oocytes are retrieved before the endometrium is fully estrogenised. The progesterone support from the corpus luteum is insufficient and can compromise endometrial receptivity. This asynchronous development between the embryo and the endometrium may explain the poor implantation rates in fresh transfer IVM cycles [95].

The endometrial preparation protocol proposed by Trounson et al. [85] included low dose exogenous estradiol starting after oocyte retrieval followed by progesterone suppositories after the embryo transfer. In a retrospective study, Elizur et al. [96] compared low-dose hMG to micronized estrogen (6 to 12 mg/day) to thicken the endometrial lining in IVM cycles when the endometrial thickness was below 6 mm on day 6–10. They observed that both hMG and micronized estrogen treatmentsignificantly improved endometrial thickness but hMG treatment was associated with higher numbers of in-vivo matured oocytes found at oocyte retrieval and higher maturation rates at 24 h. Although higher implantation and clinical pregnancy rates were observed in the hMG group, the difference was not statistically significant.

Russell et al. [96] reported improved oocyte maturation rates and embryo development when exogenous estrogen priming was initiated in the mid-follicular phase versus when estrogen was initiated early in the follicular phase. In order to mimic the natural estrogen rise from the dominant follicle, other authors have described beginning estrogen supplementation either on the day of oocyte retrieval or just before collection [97]. Progesterone supplementation is usually commenced on the day of oocyte collection to correspond to the rise of progesterone following the LH surge in natural cycles. Further studies are required to assess the optimal regimen to synchronize the window of implantation with embryo development in IVM cycles.

### Freeze all strategy

In the majority of IVM treatment cycles, IVM of immature oocytes is usually followed by a fresh ET. Several studies have shown no significant difference in clinical outcomes between fresh and frozen ETs in hCG-primed cycles, whether performed at the cleavage or blastocyst stage [92, 93]. Walls [94] also reported favourable clinical outcomes after transferring fresh blastocysts generated from FSH-primed IVM cycles, using an estradiol supplementation regime starting two days before oocyte retrieval.

However, it has been suggested that the endometrial steroid expression in non-hCG IVM cycles is abnormal, and the mid-luteal histological signature of endometrial receptivity is deficient, possibly due to the short follicular phase of IVM cycles [98]. A freeze-all approach at cleavage stages has therefore been recommended by some authors.

In a retrospective case series of 79 consecutive PCOS patients undergoing IVM followed by vitrified-warmed ET at cleavage stage over a 2-year period, the cumulative live birth rate (LBR) per embryo transfer was 16.2%, the cumulative LBR per patient was 21.8% and the LBR per retrieved immature oocyte was 1.1% [99].

In a recent randomized controlled pilot study, 40 women aged 18–37 years with a high antral follicle count undergoing one cycle of CAPA-IVM were randomized to a freeze-only strategy with subsequent frozen embryo transfer or to fresh embryo transfer at the cleavage stage. The authors reported that the ongoing pregnancy rate in the freeze-only group was significantly higher than that in the fresh embryo transfer group as was the live birth rate [100]. However, more research is needed into whether a freeze-only approach in CAPA-IVM cycles followed by a frozen embryo transfer may be a more effective and safer option in patients undergoing IVM.

### Applications of IVM in clinical practice

#### IVM for PCOS patients

IVM of oocytes has been proposed as a safer alternative to conventional ovarian stimulation (COS) in patients with PCOS as the risk of ovarian hyperstimulation syndrome (OHSS) is minimal. In a study comparing IVM versus IVF with the GnRH antagonist protocol for women with PCOS, Das et al. [8] reported that the number of mature oocytes, fertilization rates and number of embryos cleaved were similar. There was no significant difference in the clinical pregnancy rates per embryo transfer (IVF: 45.8% versus IVM: 32.4%), but the live-birth rate was higher in the IVF group (IVF: 40.7% versus IVM: 23.5%; *P* = 0.04). Five women developed moderate or severe OHSS in the IVF group, whereas none did in the IVM group. The authors concluded both IVM and IVF with the GnRH-antagonist protocol seemed to be effective treatment regimens in women with PCOS, although IVM is associated with a lower risk of OHSS.

A non-inferiority randomized controlled trial comparing IVM versus standard IVF in women with PCOS, found that one cycle of IVM without gonadotropins, resulted in lower 6-month cumulative live birth rates, when undergoing single vitrified-warmed blastocyst transfer. In the IVM group, there were no cases of OHSS, while in the IVF group, ten women (5.7%) had moderate OHSS, and one woman (0.6%) had severe OHSS [101].

A recent systematic review of prospective studies comparing IVM and conventional ovarian stimulation (COS) in patients with PCOS, found that the live birth rate was not significantly lower after IVM vs. COS (odds ratio [95% confidence interval] of 0.56 [0.32–1.01] overall, 0.83 [0.63–1.10] for human chorionic gonadotropin (hCG)-triggered IVM [hCG-IVM] and 0.45 [0.18–1.13] for non-hCG-triggered IVM [non-hCG-IVM]), irrespective of the stage of transferred embryos [102].

In recent years, strategies such as GnRH agonist triggering in combination with a policy of freeze-all embryos have been developed to reduce the risk of OHSS risk [103]. As a result, the popularity of IVM of oocytes to treat subfertile women with PCOS has declined. However, a recent study found that in women with increased risk of OHSS, women were willing to trade off cancellation rate, number of injections, chance of pregnancy and costs for lower risk of OHSS [104]. This suggests that IVM may be a suitable alternative in selected patients with PCOS after appropriate counselling.

#### IVM for fertility preservation

Time to cancer treatment is a critical concern for many cancer patients, as they often cannot afford to delay starting chemotherapy, radiation therapy, or surgery. In such urgent situations, IVM treatment offers a valuable advantage over conventional IVF. IVM can be initiated immediately and at any phase of the menstrual cycle without the need for hormonal stimulation. This allows cancer patients to preserve their fertility without having to delay cancer treatment, as the IVM treatment cycle can be completed within a short-time frame of 2–10 days [37, 105–108]. Since IVM oocyte cryopreservation can be undertaken without any need for gonadotropin stimulation, potential side effects such as OHSS can be avoided.

IVM is also a viable option in patients who have absolute contraindications to gonadotropin stimulation. Moreover, ovarian stimulation is not an option in prepubertal girls [10].

Grynberg et al. [109] observed that in breast cancer patients undergoing urgent fertility preservation, there were no differences in the number of COCs recovered or their IVM rates whatever the phase of the cycle at which oocytes were collected.

In support of these findings, Creux et al. [107] evaluated the efficacy of IVM when immature oocyte retrieval was performed in the early follicular, late follicular, or luteal phases in cancer patients undergoing urgent fertility preservation. There was no significant difference in the number of oocytes retrieved, maturation rates after 48 h of culture, fertilization rates, or the total number of oocytes and embryos cryopreserved when immature oocyte retrieval was performed at various times in the menstrual cycle.

It has been proposed that an antral follicle count of > 20 follicles and serum AMH values of 3.7 ng/ml are required for obtaining at least 10 IVM oocytes for cryopreservation in cancer patients seeking fertility preservation [110]. A second IVM retrieval 10 days after the first one may be considered if time permits. In 17 women with breast cancer who underwent 2 cycles of IVM followed by oocyte vitrification, no difference was observed between the first and second IVM outcomes, and the number of cryopreserved oocytes were comparable [111].

IVM techniques can also be combined with ovarian tissue cryobanking for urgent fertility preservation. Retrieval of immature oocytes from antral follicles extracted from excised ovarian tissue, can be combined with IVM of oocytes followed by cryopreservation of either mature oocytes or embryos [112]. Segers et al. [113] recently reported three live births after ovarian tissue oocyte IVM, intra-cytoplasmic sperm injection (ICSI) and embryo transfer among patients who underwent unilateral oophorectomy for ovarian tissue cryopreservation.

In the future, it is likely that the application of IVM procedures will expand to include conditions such as thalassemia, sickle cell anaemia and Turner syndrome [76]. In a recent study, it was observed that the biphasic in vitro maturation system (CAPA-IVM) improved the developmental competence of ovarian tissue oocytes from patients with gynaecological tumours in comparison to the standard IVM method [114].

Several studies have indicated that vitrified-warmed IVM oocytes exhibit reduced survival rates and less favourable embryological as well as clinical outcomes compared with fresh IVM oocytes [108, 115]. To enhance the viability and developmental potential of cryopreserved oocytes obtained through IVM cycles, additional research is warranted. This pursuit of improved survival and embryo development holds the potential to broaden the scope of IVM techniques for the purpose of fertility preservation.

#### IVM for resistant ovary syndrome and oocyte maturation disorders

Resistant ovary syndrome, which is also known as ovarian insensitivity syndromeor Savage syndrome, is a rare endocrine disorder characterized by elevated endogenous gonadotropin levels and low estrogen levels, primary or secondary amenorrhoea, normal secondary sexual characteristics, normal AMH and antral follicle counts, and a normal female karyotype. Mutations in the FSH receptor or beta subunit, deficiency of follicle-stimulating growth factors, abnormal gonadotropin signalling, and autoimmune abnormalities have all been described as probable causes of this disorder [116, 117]. The antral follicles are unresponsive to endogenous and exogenous FSH, and patients may therefore suffer from repeated IVF failure.

For patients with resistant ovary syndrome, IVM is currently the only viable alternative to egg donation [118]. Several live births have been reported following IVM cycles in patients with this condition [5, 118, 119]. Galvao et al. [5] observed an overall maturation rate of 27.5% after non-hCG triggered IVM and a maturation rate of 44.4% in hCG-triggered IVM cycles in patients with resistant ovary syndrome, with an overall maturation rate of 29.7%. They reported a live birth rate of 16.7% per cycle started and 33% per patient. IVM of oocytes is therefore a feasible option in patients with resistant ovary syndrome.

However, in patients with deficient oocyte maturation disorders, researchers have reported disappointing results after IVM treatment, even with extended oocyte culture [5, 120].

#### Natural cycle IVF/IVM

The concept of natural cycle IVF combined with IVM has been proposed, which combines a natural cycle IVF with immature oocyte retrieval and IVM. It has been suggested that this increases the number of embryos available for transfer, thereby increasing the possibility of a pregnancy. In a bovine model, the maturational and developmental competence of immature oocytes obtained from small antral follicles was not affected by the presence of a dominant follicle or the phase of folliculogenesis [121]. Son et al. [122] reported the results from natural cycle IVF/IVM cycles using an hCG trigger of 10,000 IU, when the diameter of the dominant follicle was over 12 mm. They reported a clinical pregnancy and implantation rate of 20.8% and 6.7% respectively. However, they concluded that although immature oocytes from natural cycle IVF can fertilize normally, the embryos derived from the immature oocytes in natural cycles IVF have a poorer reproductive potential, which suggests that embryos derived from sibling immature oocytes have little effect on the clinical outcome.

Similarly, a recent study on natural cycle IVF/IVM concluded that a significant portion of the COCs from subordinate follicles have the capacity to develop into normal embryos. The outcome was not influenced by the retrievalof a dominant follicle (12–14 mm diameter) and the developmental and implantation potential of immature oocytes retrieved from the smaller follicles were not affected [123].

In a retrospective cohort study of 1,072 patients, Teramoto et al. [124] compared the efficacy and safety of blastocyst transfers derived from small follicles (≤ 10 mm) and large follicles (> 11 mm). They observed that the incidence of abnormal karyotypes and major congenital anomalies in neonates did not differ between small and large follicle derived pregnancies.

#### IVM for poor responders

The optimum management of women who respond poorly to conventional ovarian stimulation remains a challenge. There is a paucity of data on the use of IVM protocols for poor responders. A few researchers have analyzed whether embryo transfers with rescue IVM derived embryos could improve clinical outcomes in poor-responder patients undergoing ovarian stimulation. In a case report, Liu et al. [125] reported three pregnancies (two live births and an ongoing pregnancy) in 8 poor responder patients who underwent in vitro maturation of immature oocytes derived from stimulated IVF cycles before cycle cancellation. In another study which included 440 poor responder patients, undergoing ICSI cycles in which fewer than five MII oocytes and at least one immature oocyte was retrieved, patients were divided into two groups based on the injected oocytes' nuclear maturation status. The group where only embryos derived from mature oocytes were injected were compared with cycles in which least one immature oocyte remained in culture for spontaneous maturation and ICSI. Although the rescue IVM group had a higher number of transferred embryos and a lower embryo transfer cancellation rate, there were no significant differences in the clinical pregnancy rate (16.7% vs. 16.5%) or miscarriage rate between the two groups, suggesting that rescue IVM did not provide any additional benefit in poor responder cycles [126].

Some researchers have suggested that natural cycle IVF/IVM may achieve better outcomes in poor responder patients after failure of stimulated cycles [127]. In a case report, the authors described three pregnancies in poor responders by combining natural cycle IVF with IVM of immature oocytes and suggested that natural cycle IVF/IVM could be a viable alternative for poor responder women when conventional ovarian stimulation cycles have been unsuccessful. They argued that as more oocytes are retrieved in natural cycle IVF/IVM cycles compared with natural cycle IVF, this technique could maximise treatment efficacy.

Clearly more research is needed on the optimum IVM treatment protocols and culture methods to improve clinical outcomes in poor responder patients.

#### Long term safety of IVM

Obstetric and perinatal outcomes, including Apgar scores, growth restriction, preterm delivery, neonatal complications,and pregnancy complications such as gestational diabetes and antepartum haemorrhage, are comparable in pregnancies conceived after IVF or IVM. [66, 128]. Moreover, data do not suggest a higher incidence of congenital abnormalities following IVM procedures. Among 432 children born from 344 pregnancies after ART, the observed odds ratios (ORs) for any congenital abnormality were 1.42 (95% confidence interval [CI] 0.52–3.91) for IVM, 1.21 (95% CI 0.63–2.62) for IVF, and 1.69 (95% CI 0.88–3.26) for ICSI [128]. Similarly, a study comparing 21 IVM children and 21 non-IVM children, did not report any major malformation in either group. The children born following IVM, did not show any developmental delay during infancy or childhood [129].

Very few live births have been reported following IVM oocyte cryopreservation. Cohen et al. [115] reported five live births after vitrification and warming of oocytes matured in vitro in women diagnosed with polycystic ovary syndrome. Mayeur et al. [130] recently reported three live births in cancer patients who underwent fertility preservation using IVM, two of which were from frozen oocytes and one following embryo cryopreservation.

Concerns have been raised regarding epigenetic abnormalities of oocytes or embryos generated using IVM techniques. It is reassuring that no significant imprinting gene disorders have been found in either IVM oocytes or chorionic villus or cord blood samples from newborn following IVM [131, 132].

### Improving human IVM: key challenges

While IVM presents several advantages over conventional IVF, it is important to recognize that IVM also comes with its challenges. Performing an IVM cycle demands more time and expertise compared to conventional IVF cycles [34]. Additionally, the overall efficiency of IVM tends to be lower than that of IVF cycles.

In various non-human mammalian species, assisted reproductive techniques (ART) involve the acquisition of immature GV oocytes from small antral follicles. This procedure entails visually identifying antral follicles within the ovaries and subsequently retrieving them from outside the body. This process parallels the aspiration of immature oocytes from ex vivo ovarian tissue in humans. However, in standard human IVM programs, the procedure for obtaining immature oocytes closely resembles that of standard IVF. As previously mentioned, this can present challenges for clinicians in terms of visualizing and aspirating immature oocytes during ultrasound-guided retrieval.

Furthermore, various pre-treatment methods, such as FSH- or hCG-priming before oocyte retrieval, have been employed. As a result, the techniques employed in clinical IVM can display considerable variability across different clinics. These variations in protocols and approaches can impact the effectiveness and success rates of IVM cycles, hindering the establishment of a standardized procedure.

Addressing these challenges and refining the oocyte retrieval process within IVM cycles will play a pivotal role in enhancing the overall efficiency and success rates of IVM programs. By devising specialized techniques and protocols tailored to IVM oocyte retrieval, clinicians can enhance the outcomes of IVM programs.

For embryologists, handling an IVM cycle requires additional effort compared to conventional IVF due to the following reasons:

1. Identifying COCs in follicular aspirates at the time of collection takes more time since immature oocytes lack the typical expanded CCs observed in IVF cycles (Fig. 1A). Additionally, COCs have a similar colour to granulosa cells, making it more challenging to distinguish immature oocytes from other cell types in the follicular fluid.
2. As immature oocytes are retrieved from different stages of small antral follicles, the IVM of the oocytes is not synchronized. This lack of synchronization means that multiple rounds of maturation assessment and ICSI may be necessary to obtain more mature oocytes and embryos. Achieving uniform maturation among the retrieved oocytes is crucial for successful IVM outcomes.
3. Another challenge in IVM cycles is the absence of standardized commercial media and consensus protocols for media preparation. As a result, each laboratory must prepare its own culture medium, leading to variations in the composition of the medium between different laboratories. Therefore, ongoing research is necessary to identify critical factors that influence the quantity and quality of oocyte maturation. These factors encompass the composition of the culture medium, the timing and duration of culture, as well as the presence of supportive factors. Through the identification and optimization these elements, standardized and effective culture conditions for IVM can be established.

Advanced culture methodologies, exemplified by systems like the "CAPA-IVM" biphasic approach [66], Micro-Vibration culture [133], or the utilization of small droplet (25 μL) cultures [76], along with the supplementation of various factors aimed at mimicking the in vivo follicular microenvironment, have emerged as promising avenues for future research. Furthermore, a comprehensive consideration of the physical aspects of culture systems, such as 3-D culture systems,is of considerable importance [134, 135]. These strategies have the potential to offer invaluable insights for enhancing human IVM programs in the future.

4) Reproductive scientists face challenges in improving the IVM system using immature oocytes from regular IVM cycles due to the limited number of oocytes retrieved, hindering meaningful clinical testing, and the variability in follicle diameters leading to inconsistent results. Animal studies offer advantages for researching and enhancing IVM, as they provide an ample number of oocytes for research purposes and offer a more manageable research environment. Thus, the utilization of immature oocytes from alternative sources, such as the medullaor/and CS, for research purposes in IVM programs holds the potential to provide valuable insights.

## Conclusions

In the field of assisted reproduction and fertility preservation, IVM of human immature oocytes continues to be a relevant and valuable technique. IVM offers several advantages and potential benefits, making it a valuable choice for specific individuals or clinical situations, such as those seeking fertility preservation, at risk of OHSS, or facing economic constraints in pursuing conventional IVF treatment. Additionally, IVM's contributions extend to the wider field of reproductive medicine. By studying oocyte maturation, valuable insights can be gained, potentially advancing infertility treatment, optimizing embryo development, and ultimately leading to improved clinical outcomes. To fully unlock the potential of IVM and ensure its widespread utilization, collaborative efforts among clinicians, scientists, embryologists, and industry professionals are essential. By working together, these experts can address the challenges, refine laboratory techniques, and develop standardized protocols that enhance the efficiency and effectiveness of IVM programs. IVM can serve as a valuable alternative, particularly for individuals who cannot undergo COH. With enhanced efficiency, IVM is likely to become increasingly relevant and beneficial in routine clinical practice, providing more options for individuals seeking fertility treatments and fertility preservation.

## Data Availability

Not applicable.
